# Incidence, Risk Factors, and Treatment of Autoimmune Cytopenia Following Pediatric Allogeneic Hematopoietic Stem Cell Transplantation

**DOI:** 10.1155/joot/3676059

**Published:** 2025-12-11

**Authors:** Changlan Chen, Yingying Wang, Yan Meng, Ying Dou, Luying Zhang, Xianmin Guan, Xiaoying Lei, Jie Yu

**Affiliations:** ^1^ Department of Pediatrics, The First Affiliated Hospital of Xi’an Jiaotong University, Xi’an, China, xjtu.edu.cn; ^2^ Department of Hematology Oncology Children’s Hospital of Chongqing Medical University, National Clinical Research Center for Child Health and Disorders, Ministry of Education Key Laboratory of Child Development and Disorders, Chongqing Engineering Research Center of Stem Cell Therapy, Chongqing, 400014, China, moe.edu.cn

**Keywords:** allogeneic hematopoietic stem cell transplantation, autoimmune cytopenia, chronic graft-versus-host disease, pediatric

## Abstract

Autoimmune cytopenia (AIC) following pediatric allogeneic hematopoietic stem cell transplantation (allo‐HSCT) is relatively rare but it is a challenging complication, and standardized treatment guidelines are lacking. We retrospectively analyzed 436 pediatric patients undergoing allo‐HSCT; 37 (8.5%) developed AIC, characterized by autoimmune hemolytic anemia (*n* = 13), immune thrombocytopenia (*n* = 11), and Evans syndrome (*n* = 13). Risk factor analysis revealed that younger age at HSCT, nonmalignant diseases, unrelated donor transplantation, and chronic graft‐versus‐host disease (cGVHD) were significantly associated with the development of AIC. Through multivariate analysis, cGVHD was identified as an independent risk factor for AIC. In our study, the first‐line treatment for AIC involved steroids and/or intravenous immunoglobulin, with a complete remission rate of 48.6%. Additional therapeutic strategies included rituximab, which led to complete remission in 5 of 12 patients we treated, and sirolimus, with 3 of 7 patients achieving complete remission. Three patients achieved partial remission, while 9 patients died due to complications, such as severe infections, extensive GVHD, and multiorgan bleeding. Our findings suggest that cGVHD is an independent risk factor for post‐transplant AIC and is typically associated with adverse outcomes, highlighting the critical importance of timely and effective interventions.

## 1. Introduction

Autoimmune cytopenia (AIC) following allogeneic hematopoietic stem cell transplantation (allo‐HSCT) is a relatively rare yet serious complication, characterized by the destruction of donor‐derived blood cells mediated by the donor’s immune system, leading to singular or multilineage cytopenia. Autoimmune hemolytic anemia (AIHA) is the predominant manifestation, followed by immune thrombocytopenia (ITP), Evans’ syndrome, and autoimmune neutropenia (AIN). AIC predominantly occurs within 5–10 months post‐HSCT, with an incidence of 5%–10% in pediatric patients [1–6], and surging up to 20% in specific nonmalignant diseases [7, 8]. Reported risk factors include younger age at transplantation, nonmalignant diseases, unrelated donor transplantation, cord blood transplantation, the use of lymphocyte‐depleting agents in the conditioning regimen, chronic graft‐versus‐host disease (cGVHD), and viral infection [5, 9–13]. Underlying mechanisms may be rooted in incomplete immune reconstitution or immune dysregulation post‐HSCT. This therapeutic approach for post‐HSCT AIC predominantly aligns with strategies established for primary AIHA. First‐line treatment includes steroids and/or intravenous immunoglobulin (IVIG), yielding remission in a majority of patients [5, 14]. However, recurrence rates are notably elevated, particularly upon tapering steroids. Additional treatment options include rituximab (RTX), sirolimus [2, 4, 5, 10], and newer immunosuppressive therapies, such as bortezomib [15, 16], daratumumab [17, 18], and abatacept [19], which have shown promising results in some patients.

The management of post‐transplant AIC remains challenging, as responses to immunosuppressive therapy are often incomplete or nondurable [4]. In addition, post‐transplant AIC is often associated with other complications, such as immunodeficiency, infections, and GVHD. The concurrence of these conditions not only increases diagnostic and therapeutic complexity but also exacerbates immune dysregulation, leading to a significantly increased risk of mortality [9, 20]. Despite the introduction of targeted biological agents and supportive care advances, the prognosis for severe or refractory post‐transplant AIC remains unsatisfactory [15, 17], highlighting the urgent need for more effective therapeutic strategies.

This retrospective study examined clinical data from 436 pediatric patients undergoing allo‐HSCT at the Hematology‐Oncology Center of Chongqing Medical University Affiliated Children’s Hospital between January 2014 and December 2021. It aimed to assess the incidence and risk factors and describe treatment outcomes for patients with post‐transplant AIC. The findings may provide guidance for the management and treatment of refractory/recurrent AIC.

## 2. Patients and Methods

### 2.1. Patients

This study was approved by the Institutional Review Board of Children’s Hospital of Chongqing Medical University (ethics approval number: 2023 Lunshen [Research] No. 36). Because this is a retrospective study, the need for patient consent for data collection was waived by the Institutional Review Board. This study involved 436 pediatric patients who underwent allo‐HSCT at the Hematology‐Oncology Center of Chongqing Medical University Affiliated Children’s Hospital from January 2014 to December 2021. The patients were categorized into two groups: one with malignant diseases, such as acute leukemia and myelodysplastic syndrome, and the other with nonmalignant diseases, including severe thalassemia, severe aplastic anemia, lymphoproliferative diseases, and primary immunodeficiency diseases (e.g., Wiskott–Aldrich syndrome, chronic granulomatous disease, severe combined immunodeficiency, hyper‐IgM syndrome, and others). Patients who underwent allo‐HSCT and subsequently developed AIC were included for analysis. Before transplantation, all patients were screened using the direct antiglobulin test (DAT). Patients with DAT positivity due to ABO antibodies, history of AIHA, or positive DAT results before HSCT were excluded from our study. Conditioning regimens are tailored as either myeloablative or reduced intensity based on the primary disease. Post‐transplantation, GVHD prophylaxis included cyclosporine, mycophenolate mofetil, and/or methotrexate. All patients received IVIG support therapy initiated on day 7 post‐transplant at a dose of 0.4–0.5 g/kg weekly, followed by monthly infusions during outpatient follow‐up for approximately 1 year, until immune reconstitution was achieved.

### 2.2. AIC Definitions and Diagnosis

AIHA was diagnosed based on the following criteria: (1) hemoglobin < 100 g/L (or a significant decrease from baseline) with a reticulocyte count > 120 × 10^9^/L or > 3%; (2) laboratory evidence of hemolysis, including lactate dehydrogenase > 1.5 × upper limit of normal (ULN), total bilirubin > 1.5 × ULN, and haptoglobin < 0.3 g/L; (3) positive DAT; and (4) exclusion of patients with DAT positivity mediated by ABO antibodies, a history of AIHA, or prior DAT positivity before allo‐HSCT, as well as other causes of immune hemolytic anemia. ITP and AIN pertain to unexplained isolated thrombocytopenia or neutropenia, defined by a platelet count < 100 ∗ 10^9^/L and a neutrophil count < 1.0 ∗ 10^9^/L, respectively. Other potential causes of cytopenia, such as drug toxicity, GVHD, or underlying viral infections following HSCT, were ruled out. In appropriate patients, bone marrow biopsy was performed to exclude cytopenia attributed to bone marrow insufficiency. Evans syndrome refers to the simultaneous or sequential occurrence of cytopenia involving two or three cell lineages.

### 2.3. Treatments

In our study, first‐line therapy for AIC consisted of steroids, initiated at 4–6 mg/kg/day and escalated to 10–20 mg/kg/day in severe cases as clinically indicated, followed by gradual tapering according to therapeutic response. Adjunctive interventions, including IVIG (0.5–1 g/kg/day), transfusion support, and other supportive measures, were applied based on clinical need. Patients refractory to first‐line therapy received rituximab at 375 mg/m^2^ weekly for 4–6 doses. Sirolimus, at 0.5–2 mg/day, was subsequently introduced if additional immunosuppression was required. In patients with ITP, thrombopoietin was administered to enhance platelet production. Further therapy adjustments were guided by patient response and the discretion of the attending physicians.

Treatment response was defined as follows: complete response, normalization of hemoglobin (age‐adjusted for children), a platelet count > 100 × 10^9^/L, and a neutrophil count > 1.5 × 10^9^/L, accompanied by normalization of hemolytic markers and sustained transfusion independence for at least 2 months; partial response, hemoglobin increase > 2 g/dL without transfusion in the preceding 7 days, accompanied by improvement in hemolytic markers, a platelet count at least twice the pretreatment level without bleeding, and a neutrophil count > 1.0 × 10^9^/L, with continued therapy to maintain the response; and nonresponse, unchanged or worsening manifestations despite treatment.

### 2.4. Post‐HSCT Monitoring Parameters

For patients undergoing allo‐HSCT, cytomegalovirus (CMV) and Epstein–Barr virus (EBV) loads were monitored weekly during the first 2 months post‐HSCT using quantitative polymerase chain reaction (PCR). Subsequently, monitoring continued during all scheduled outpatient clinic visits. The diagnostic thresholds for viral viremia were defined as CMV > 1000 copies/mL and EBV > 2000 copies/mL.

Chimerism analysis was performed on days +14 after HSCT and continued monthly until stabilization, using short tandem repeat (STR)–PCR technology. Complete chimerism was defined as > 95% of donor chimerism in peripheral blood. Mixed chimerism was defined as between 5% and 95% of donor chimerism in peripheral blood, or in one or more lineage. Graft failure was characterized by donor chimerism < 5%. Cases of early mortality post‐HSCT were categorized under unassessed STR status due to the inability to conduct subsequent evaluations.

### 2.5. Statistical Analysis

Baseline characteristics of the study cohort were summarized using descriptive statistics. Quantitative variables, represented as median and range, underwent univariate analysis through analysis of the Mann–Whitney *U* test. Categorical variables were presented as absolute number and percentage, and univariate analysis was performed using the chi‐square test or Fisher’s exact test. The following variables were included in the univariate analysis of factors predicting the development of AIC: gender, primary diseases, median age at HSCT, donor type, stem cell source, the use of ATG serotherapy, ABO and HLA compatibility, acute and chronic GVHD, CMV or EBV viremia, and chimerism status at the time of AIC diagnosis. Variables with *p* < 0.05 in univariate analysis were subjected to multivariate logistic regression analysis to identify independent risk factors for post‐HSCT AIC. The endpoints were the incidence of AIC and treatment response. Statistical analyses were performed using the SPSS software Version 25.0. All *p* values were 2‐sided, and *p* < 0.05 was considered statistically significant. *p* > 0.1 was reported as not significant, whereas *p* between 0.05 and 0.1 was reported in detail.

## 3. Results

### 3.1. Patients

In this study, a total of 436 patients underwent allo‐HSCT, with a median age at transplantation of 3.3 years (range: 0.3–17.4 years) and a median follow‐up duration of 21.8 months (range: 0.1–102.4 months). Among them, 37 patients (8.5%) developed post‐transplant AIC, including AIHA (*n* = 13), ITP (*n* = 11), and Evans syndrome (*n* = 13, including 8 AIHA + ITP, 1 AIN + AIHA, 1 AIN + ITP, and 3 AIHA + ITP + AIN). No isolated AIN was observed. Cumulative incidence of post‐transplant AIC is shown in Figure 1. The median age at onset of post‐transplant AIC was 2.6 years (range: 1.0–17.6 years), and the median time to onset was 147 days post‐transplant (range: 22–652 days). Detailed baseline HSCT characteristics of all patients are summarized in Table 1.

Figure 1Cumulative incidence of post‐transplant AIC. (a) All patients; (b) patients with malignant disease versus patients with nonmalignant disease. (c) Patients with sibling donor versus patients with unrelated donor versus patients with haploidentical donor; (d) patients with cGVHD versus patients without cGVHD.

**Figure:**
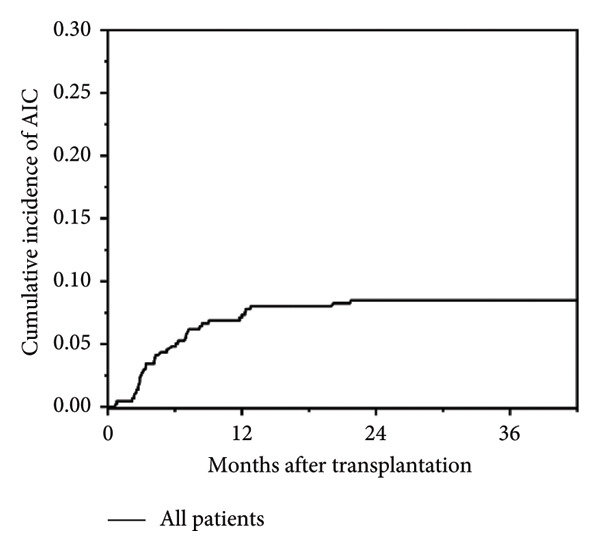
(a)

**Figure:**
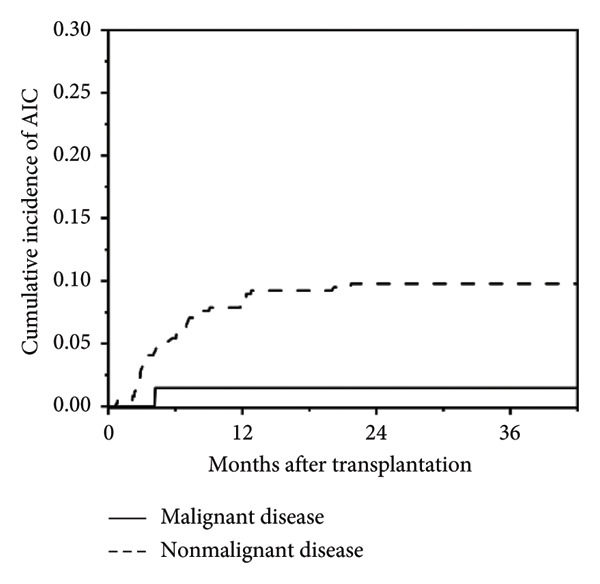
(b)

**Figure:**
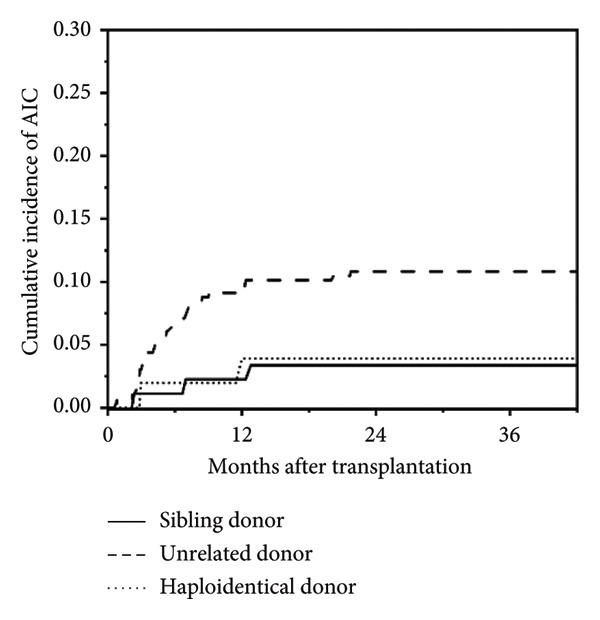
(c)

**Figure:**
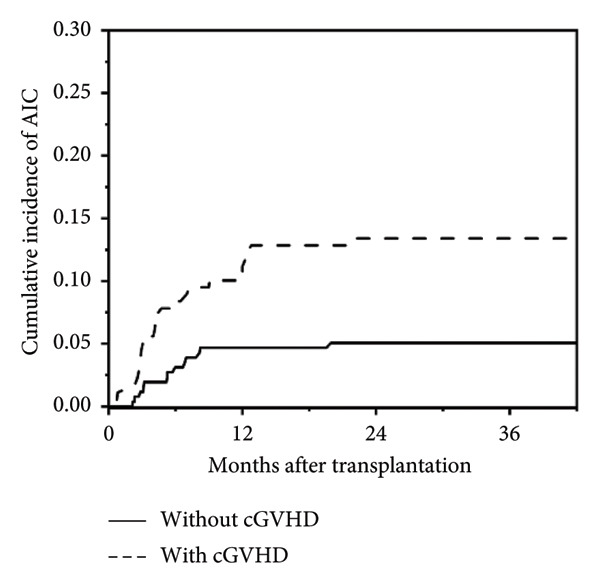
(d)

**Table 1:** Patient and transplant characteristics.

Characteristics	Patients with AIC	Patients without AIC	*p* value
No. of patients	37	399	
Median age at HSCT, year (range)	2.1 (0.7–17.4)	3.3 (0.3–14.8)	0.028
Gender			
Male	29 (78.4)	276 (69.2)	0.243
Female	8 (21.6)	123 (30.8)	
Diagnosis			
Malignant	1 (2.7)	67 (16.8)	0.024
Nonmalignant	36 (97.3)	332 (83.2)	
Donor type			
Sibling donor	3 (8.1)	86 (21.6)	0.040
Unrelated donor	32 (86.5)	264 (66.2)	
Haploidentical donor	2 (5.4)	49 (12.3)	
Stem cell source			
Bone marrow^∗^	0	14 (3.5)	0.105
Peripheral blood	33 (89.2)	369 (92.5)	
Cord blood	4 (10.8)	16 (4.0)	
Serotherapy			
ATG	35 (94.6)	331 (83.0)	0.065
No ATG	2 (5.4)	68 (17.0)	
HLA match			
10/10	17 (45.9)	196 (49.1)	0.712
≤ 9/10	20 (54.1)	203 (50.9)	
ABO donor/recipient			
Identical	11 (29.7)	158 (39.6)	0.358
Compatible (donor O+)	6 (16.2)	73 (18.3)	
Other	20 (54.1)	168 (42.1)	
CMV/EBV viremia			
EBV	12 (32.4)	107 (26.8)	0.800
CMV	1 (2.7)	27 (6.8)	
Both	20 (54.1)	222 (55.6)	
Acute GVHD, any	30 (81.1)	285 (71.4)	0.210
Grades I‐II	26 (70.3)	238 (59.6)	
Grades III‐IV	4 (10.8)	47 (11.8)	
Chronic GVHD, any	24 (64.9)	155 (38.8)	< 0.001
Limited	17 (46.0)	136 (34.1)	
Extensive	7 (18.9)	19 (4.7)	
Chimerism			
Full	32 (86.5)	344 (86.2)	0.321
Mixed	5 (13.5)	29 (7.3)	
Graft failure	0	14 (3.5)	
untested^#^	0	12 (3.0)	

*Note:* Values are no. (%) unless otherwise indicated. CMV, cytomegalovirus.

Abbreviations: ATG, antithymocyte globulin; EBV, Epstein–Barr virus.

^∗^Three underwent CB + BM transplantation.

^#^STR analysis was not performed due to early post‐transplant mortality.

### 3.2. Risk Factors

Univariate analysis showed no statistically significant differences between patients with and without AIC in terms of gender, stem cell source, ATG serotherapy, HLA and ABO compatibility, EBV/CMV viremia, chimerism status, or acute GVHD occurrence. However, significant associations with AIC were revealed in the following variables: younger age at HSCT (2.1 years [range, 0.7–17.4] in patients with AIC vs. 3.3 years [range, 0.3–14.8 years] in those without AIC; *p* = 0.028), primary diagnosis of a nonmalignant disease (*p* = 0.024), unrelated donor transplantation (*p* = 0.040), and concurrent cGVHD (*p* < 0.001). Multivariate analysis further delineated cGVHD as the only independent risk factor for AIC development post‐HSCT, as detailed in Table 2.

**Table 2:** Multivariate analysis of risk factors associated with the development of AIC.

Risk factors	Odds ratio	95% CI	*p* value
Age at HSCT (year)	0.881	0.769–1.010	0.069
Diagnosis			
Nonmalignant vs. malignant	5.465	0.706–42.277	0.104
Donor			
URD vs SD	2.742	0.799–9.414	0.109
HID vs SD	0.666	0.098–4.548	0.679
cGVHD			
Limited vs. absent	2.392	1.110–5.156	0.026
Extensive vs. absent	9.868	3.161–30.811	0.000

Abbreviations: HID, haploidentical donor; SD, sibling donor; URD, unrelated donor.

### 3.3. Treatment and Outcomes

Transplant characteristics for AIC patients are summarized in Table 3, and the treatment and outcomes for these individuals are detailed in Table 4. All patients received steroids and/or IVIG as first‐line treatment, supplemented with transfusion therapy and plasma exchange as clinically required, achieving a complete remission rate of 48.6% (18/37). Other second‐line or more advanced therapeutic approaches involved RTX (*n* = 12), sirolimus (*n* = 7), mycophenolate mofetil (*n* = 5), thrombopoietin (*n* = 1), and cyclophosphamide (*n* = 1). Of these, RTX achieved complete remission in 5 of the 12 patients treated. Sirolimus resulted in complete remission for 3 of 7 patients, and thrombopoietin successfully induced complete remission in the single patient treated. Overall, 27 patients (73.0%) reached complete remission, with a median remission duration of 86 days (range, 15–931 days). Additionally, 3 patients (8.1%) experienced partial remission.

**Table 3:** Clinical characteristics of patients with AIC.

No.	Diagnosis	Sex	Age at HSCT, yr(s)	Age at AIC, yr(s)	HSCT type			Chimerism status	aGVHD maximum grade	cGVHD	AIC
					Donor	Stem source	Conditioning regimen				
1	ALL	F	2.1	2.4	MMUD	CB	BU + CY	Full	III	Limited	ITP
2	WAS	M	1.0	1.4	MUD	CB	BU + CY	Full	II	Limited	AIHA
3	Thalassemia M	M	1.2	1.8	MMUD	PB	BU + FLU + ATG	Full	II	Limited	AIHA + ITP
4	WAS	M	3.6	3.8	MMUD	CB	BU + FLU + ATG	Full	I	Limited	AIHA + ITP
5	SCID	M	17.4	17.6	MSD	PB	BU + FLU + ATG	Full	Absent	Limited	ITP
6	WAS	M	1.7	1.9	MMUD	CB	BU + FLU + ATG	Full	II	Absent	ITP
7	Thalassemia M	M	1.5	2.1	MMSD	PB	CY + BU + FLU + ATG	Full	II	Absent	AIHA
8	WAS	M	0.9	1.6	MUD	PB	BU + CY + ATG	Full	II	Absent	AIHA
9	Thalassemia M	M	1.9	3.8	MMUD	PB	CY + BU + FLU + ATG	Full	II	Limited	ITP
10	CGD	M	0.8	1.1	MUD	PB	BU + CY + ATG	Full	I	Absent	ITP
11	CGD	M	0.9	1.4	MMUD	PB	BU + CY + ATG	Full	II	Absent	AIHA + ITP
12	WAS	M	0.7	1.0	MMUD	PB	BU + CY + ATG	Mixed	II	Limited	ITP
13	Thalassemia M	M	2.0	3.0	MMUD	PB	BU + CY + FLU + ATG	Full	Absent	Limited	AIHA
14	CGD	M	0.8	1.4	MUD	PB	BU + CY + ATG	Full	I	Absent	AIHA
15	CGD	M	7.5	8.6	MSD	PB	BU + CY + ATG	Full	II	Extensive	ITP
16	CGD	M	5.0	5.2	MMUD	PB	BU + CY + ATG	Mixed	Absent	Absent	AIHA
17	WAS	M	1.6	2.5	MMUD	PB	BU + CY + ATG	Full	II	Limited	AIHA
18	HIGM	M	3.5	3.9	MUD	PB	BU + CY + ATG	Full	I	Absent	AIHA + ITP
19	HIGM	M	1.9	2.4	MUD	PB	BU + CY + ATG	Full	Absent	Absent	AIHA
20	WAS	M	0.8	1.6	MMUD	PB	BU + CY + ATG	Full	II	Extensive	AIHA + ITP
21	CGD	M	1.3	1.9	MUD	PB	BU + CY + ATG	Full	I	Absent	AIHA
22	CGD	M	2.7	3.1	MUD	PB	BU + CY + ATG	Full	I	Limited	AIHA + ITP + AIN
23	Thalassemia M	M	2.3	2.5	MUD	PB	CY + BU + FLU + ATG	Mixed	I	Absent	AIHA
24	Thalassemia M	F	2.1	3.8	MUD	PB	CY + BU + FLU + ATG	Full	Absent	Absent	AIHA
25	Thalassemia M	F	6.2	6.4	MUD	PB	CY + BU + FLU + ATG	Full	II	Absent	ITP
26	SAA	F	11.1	11.3	MUD	PB	CY + BU + FLU + ATG	Full	II	Limited	ITP
27	HIGM	M	1.4	1.7	MUD	PB	BU + CY + ATG	Full	II	Limited	AIN + AIHA
28	Thalassemia M	M	1.8	2.4	MUD	PB	CY + BU + FLU + ATG	Mixed	Absent	Limited	AIHA
29	Thalassemia M	F	3.1	3.3	MUD	PB	CY + BU + FLU + ATG	Full	I	Extensive	AIHA + ITP + AIN
30	HIGM	M	3.3	3.3	MMUD	PB	CY + BU + ATG	Full	III	Limited	AIHA + ITP
31	Thalassemia M	F	2.4	3.4	MMUD	PB	CY + BU + FLU + ATG	Full	II	Limited	ITP
32	WAS	M	6.3	6.6	MMUD	PB	CY + BU + ATG	Full	II	Extensive	ITP
33	Thalassemia M	M	4.8	5.0	MMUD	PB	CY + BU + FLU + ATG	Full	II	Limited	ITP + AIN
34	Thalassemia M	F	2.8	3.5	MMUD	PB	CY + FLU + BU + ATG	Full	II	Extensive	AIHA + ITP
35	SAA	F	4.5	4.8	HID	PB	CY + BU + FLU + ATG	Full	III	Extensive	AIHA + ITP + AIN
36	WAS	M	1.2	2.2	HID	PB	BU + CY + ATG	Mixed	Absent	Limited	AIHA
37	Thalassemia M	M	2.5	2.6	MMUD	PB	CY + FLU + BU + ATG	Full	IV	Extensive	AIHA + ITP

*Note:* M, male; F, female; Thalassemia M, thalassemia major; HIGM, hyper‐IgM syndrome; Bu, busulfan; CY, cyclophosphamide; Flu, fludarabine; yr(s), year(s).

Abbreviations: ALL, acute lymphoblastic leukemia; ATG, antithymocyte globulin; CB, cord blood; CGD, chronic granulomatous disease; HID, haploidentical donor; MMSD, mismatched sibling donor; MMUD, mismatched unrelated donor; MSD, matched sibling donor; MUD, matched unrelated donor; PB, peripheral blood; SAA, severe aplastic anemia; SCID, severe combined immunodeficiency disease; WAS, Wiskott–Aldrich syndrome.

**Table 4:** Treatment, response, and outcomes of patients with AIC.

No.	Type of AIC	Time to AIC, d	Time to CR, d	Treatment and response						Outcome
				First‐line therapy	Response	Second‐line therapy	Response	Third‐ line therapy	Response	
1	ITP	126	35	MPD	CR	N/A	N/A	N/A	N/A	Alive
2	AIHA	128	119	MPD + IVIG	CR	N/A	N/A	N/A	N/A	Alive
3	AIHA + ITP	189	341	MPD + IVIG	NR	PX ∗ 3 + CY ∗ 2 + RTX ∗ 4	CR	N/A	N/A	Alive
4	AIHA + ITP	102	133	MPD	CR	N/A	N/A	N/A	N/A	Alive
5	ITP	70	145	PDN	CR	N/A	N/A	N/A	N/A	Alive
6	ITP	74	119	PDN	CR	N/A	N/A	N/A	N/A	Alive
7	AIHA	209	68	MPD	CR	N/A	N/A	N/A	N/A	Alive
8	AIHA	253	281	MPD	NR	MPD + IVIG + RTX ∗ 4	CR	N/A	N/A	Alive
9	ITP	652	81	PDN	CR	N/A	N/A	N/A	N/A	Alive
10	ITP	96	86	MPD + IVIG	CR	N/A	N/A	N/A	N/A	Alive
11	AIHA + ITP	172	37	MPD + IVIG	CR	N/A	N/A	N/A	N/A	Alive
12	ITP	142	NR	MPD + IVIG	NR	N/A	N/A	N/A	N/A	Dead
13	AIHA	371	15	MPD	CR	N/A	N/A	N/A	N/A	Alive
14	AIHA	214	163	MPD	NR	RTX ∗ 5 + IVIG	CR	N/A	N/A	Alive
15	ITP	384	152	MPD + IVIG	CR	N/A	N/A	N/A	N/A	Alive
16	AIHA	65	273	MPD	CR	N/A	N/A	N/A	N/A	Alive
17	AIHA	354	931	MPD	NR	PDN + MMF	PR	MMF + SRL	CR	Alive
18	AIHA + ITP	158	53	MPD	CR	N/A	N/A	N/A	N/A	Alive
19	AIHA	182	31	MPD	CR	N/A	N/A	N/A	N/A	Alive
20	AIHA + ITP	272	NR	IVIG + PDN	NR	MPD + RTX ∗ 1	NR	N/A	N/A	Dead
21	AIHA	247	16	MPD + IVIG	CR	N/A	N/A	N/A	N/A	Alive
22	AIC	124	117	MPD + IVIG	NR	MPD + RTX ∗ 2	CR	N/A	N/A	Alive
23	AIHA	102	74	MPD + IVIG	NR	MPD + RTX ∗ 6	NR	SRL + MMF	CR	Alive
24	AIHA	606	PR	IVIG + PDN	PR	N/A	N/A	N/A	N/A	Alive
25	ITP	86	NR	MPD	NR	RTX ∗ 1	NR	N/A	N/A	Dead
26	ITP	77	108	MPD	CR	N/A	N/A	N/A	N/A	Alive
27	AIN + AIHA	86	NR	MPD + IVIG	NR	SRL	NR	N/A	N/A	Dead
28	AIHA	210	PR	MPD	NR	PDN + MMF	PR	N/A	N/A	Alive
29	AIC	92	PR	MPD + IVIG	NR	RTX ∗ 5	NR	SRL + MMF	PR	Dead
30	AIHA + ITP	24	105	MPD	NR	RTX ∗ 4	NR	SRL	CR	Alive
31	ITP	368	76	MPD + IVIG	CR	N/A	N/A	N/A	N/A	Alive
32	ITP	81	39	MPD + IVIG	NR	PDN + TPO	CR	N/A	N/A	Alive
33	ITP + AIN	83	NR	MPD	NR	RTX ∗ 1	NR	N/A	N/A	Dead
34	AIHA + ITP	218	NR	IVIG + PDN	NR	MMF + PDN	NR	SRL	NR	Dead
35	AIC	88	54	MPD	NR	RTX ∗ 1	CR	N/A	N/A	Dead
36	AIHA	360	71	MPD	CR	N/A	N/A	N/A	N/A	Alive
37	AIHA + ITP	22	NR	MPD	NR	RTX ∗ 4	NR	SRL	NR	Dead

*Note:* PX, plasma exchange; SRL, sirolimus; CY, cyclophosphamide; MPD, methylprednisolone; MMF, mycophenolate mofetil; IVIG, intravenous immunoglobulin; TPO, thrombopoietin; PDN, prednisone; RTX∗n, number of doses of rituximab.

Abbreviations: CR, complete response; NR, nonresponse; PR, partial response.

At the last follow‐up, 28 of the AIC patients (75.7%) were alive, while 9 (34.3%) died. Among these, one patient with AIHA, ITP, and AIN achieved partial remission but died due to a severe infection and immunological encephalitis on day 351. Another patient, despite reaching complete remission for AIHA + ITP + AIN, died due to complications of immunological encephalitis and impaired consciousness. The causes of death for the others included severe aspergillosis in 3 patients and multiorgan bleeding in 4.

## 4. Discussion

This study presents the incidence, risk factors, and treatments for AIC after allo‐HSCT. The median time to onset was 147 days post‐transplant (range: 22–652 days), with an incidence of 8.5% (37/436), aligning with findings from previous studies [1, 8, 21]. Notably, 36 of 37 patients had nonmalignant diseases, in particular they were patients with high IgM syndrome, where the incidence of AIC post‐transplantation reached 26.7% (4/15). Recent studies have indicated that nonmalignant diseases are associated with the development of AIC after transplantation. For instance, Page et al. reported a 56% incidence of AIC in children with metabolic diseases post‐transplant [22], and Kruizinga et al. found that β‐thalassemia was present in 9 of 26 post‐transplant AIC patients [10]. This trend suggests that pretransplant abnormal immune status in patients with nonmalignant diseases may elevate the risk for developing autoimmune disorders post‐transplant.

Our study indicates that risk factors for post‐transplant AIC include younger age at transplantation, nonmalignant diseases, unrelated donor transplantation, and concurrent cGVHD. Notably, cGVHD has been identified as an independent risk factor for the development of AIC. Studies, such as Lum et al., have also underscored the role of cGVHD in the development of AIC [5], suggesting that it negatively affects immune reconstitution and thymic regeneration, potentially leading to regulatory T‐cell deficiency and increased AIC susceptibility [23]. Patients with extensive gastrointestinal cGVHD warrant special attention due to the increased severity and complexity of their condition. For example, patients 20, 34, and 37 developed AIHA and ITP after HSCT, accompanied by severe gastrointestinal cGVHD, resulting in significant anemia, infections, diarrhea, and multiorgan bleeding. Even with the use of multiple drugs in combination, disease control was challenging, ultimately leading to the mortality of these 3 patients.

Additionally, existing research points to potential correlations between AIC development and factors, such as cord blood transplantation [6, 11, 14] and viral infections, notably CMV [10]. Yet, our findings do not conclusively validate these associations. This could be due to the relatively small number of patients involving cord blood transplantation (4.6%) and the high prevalence of concurrent CMV and/or EBV infections in the patients (89.2%). Despite this, careful monitoring for viral infections, particularly CMV, remains crucial due to its implicated role in immune system dysregulation [10, 24].

The underlying pathogenesis of post‐transplant AIC is not fully understood, possibly associated with incomplete immune reconstitution or immune dysregulation after transplantation. Some studies suggest that the use of immunosuppressive agents (such as alemtuzumab or ATG) pre‐HSCT to suppress T‐cell proliferation may result in an imbalance in lymphocyte subsets, potentially promoting autoimmune reactions [2, 6, 10]. Notably, in our study, 94.6% (35/37) of AIC patients received ATG serotherapy (*p* = 0.065). Among patients who underwent immune assessments at the time point closest to AIC onset, we observed that B‐cell recovery occurred more rapidly than T‐cell reconstitution in most patients, suggesting that early B‐cell predominance may contribute to immune imbalance and AIC development. A small proportion of patients, however, did not undergo complete immune profiling, which limited our ability to comprehensively evaluate the relationship between immune reconstitution and post‐transplant AIC. Nevertheless, these observations may serve as a useful guide for future research exploring the pathogenesis of AIC.

Existing research indicates that the majority of AIC patients occur in the setting of complete chimerism, suggesting that the autoantibodies against donor blood cells stem from donor plasma cells [10, 20, 25, 26]. However, the study by Even‐Or et al. revealed that 46.2% (6/13) of patients exhibited mixed chimerism at the time of AIC diagnosis, implying that residual antibody‐secreting host cells could also play a role in its pathogenesis [1]. This may potentially account for the 5 patients of mixed chimerism observed in our study.

In our study, the first‐line treatment included steroids and/or IVIG, achieving a complete remission rate of 48.6%, a higher rate compared to most reported studies [5, 14, 27]. When first‐line treatment is ineffective, second‐ or third‐line therapies, such as RTX and sirolimus, are commonly employed to suppress aberrant immune responses. Both these drugs have been proven effective in prior research [4, 5, 14]. Our study highlights that sirolimus was typically administered in the mid‐ to late stages of the disease when patients often presented with severe complications, such as severe GVHD and infections. This complexity could potentially impact the expected efficacy of sirolimus. Therefore, early consideration of combination therapies to control disease progression is crucial when managing refractory or recurrent AIC. Additional treatments employed in our study, such as mycophenolate mofetil and cyclophosphamide, have been documented in other studies [4, 11]; yet, these approaches yielded limited therapeutic efficacy.

In our study, 9 AIC patients (24.3%) died, sharing a common characteristic of abnormal immune status (such as immunodeficiency, severe GVHD, and other autoimmune disorders). Additionally, the increased risk of bleeding due to thrombocytopenia further complicated matters. The interaction of these factors made patients more susceptible to severe infections, autoimmune responses, and multiorgan bleeding. Therefore, exploring innovative therapeutic strategies is crucial for effectively managing and halting the progression of AIC. Recent studies have highlighted the efficacy of bortezomib, daratumumab, and abatacept in treating refractory or recurrent AIC [18, 19, 28–30]. These drugs could be worth considering for future treatments.

## 5. Conclusion

Our study revealed an incidence of AIC after allo‐HSCT of 8.5%, notably higher among patients with nonmalignant diseases, with concurrent cGVHD identified as a significant independent risk factor. First‐line treatment involving steroids and/or IVIG resulted in a 48.6% complete remission rate, while the follow‐up treatments, such as RTX and sirolimus, also helped some patients to achieve complete remission. Overall, the treatment of post‐transplant AIC is challenging and usually associated with poor prognosis, the severity of which may vary from individual to individual, and sometimes may even lead to death. Therefore, close monitoring of patients who may develop AIC is required when allo‐HSCT is performed, especially those with nonmalignant disease and severe cGVHD. For patients who have already developed AIC, the necessary therapeutic measures need to be taken in a timely manner to avoid adverse outcomes.

## Disclosure

All authors approved the final manuscript.

## Conflicts of Interest

The authors declare no conflicts of interest.

## Author Contributions

Changlan Chen and Jie Yu conceptualized the study. Changlan Chen, Yingying Wang, Yan Meng, and Xiaoying Lei participated in data collection and statistical analysis. Changlan Chen and Yingying Wang drafted the manuscript. Ying Dou, Luying Zhang, Xianmin Guan and Xiaoying Lei, and Jie Yu critically reviewed the draft. Changlan Chen, Yingying Wang, and Yan Meng revised the manuscript.

## Funding

The authors thank the support of the Specialized Research Project on Stem Cell Therapy (No. 30000222).

## Data Availability

The data that support the findings of this study are available from the corresponding author upon reasonable request.
